# The effect of individual and neighborhood socioeconomic status on esophageal cancer survival in working-age patients in Taiwan

**DOI:** 10.1097/MD.0000000000004140

**Published:** 2016-07-08

**Authors:** Chin-Chia Wu, Chun-Ming Chang, Ta-Wen Hsu, Cheng-Hung Lee, Jian-Han Chen, Chih-Yuan Huang, Ching-Chih Lee

**Affiliations:** a Division of Colorectal Surgery; b Division of General Surgery, Department of Surgery; c Cancer Center, Dalin Tzu Chi Hospital, Buddhist Tzu Chi Medical Foundation, Chiayi; d School of Medicine, Tzu Chi University, Hualien; e Division of Nephrology, Department of Internal Medicine, Ditmanson Medical Foundation Chia-Yi Christian Hospital, Chia-Yi; f Department of Otorhinolaryngology, Head and Neck Surgery, Kaohsiung Veterans General Hospital, Kaohsiung; g Department of Otolaryngology, Head and Neck Surgery, Tri-Service General Hospital; h School of Medicine, National Defense Medical Center, Taipei, Taiwan.

**Keywords:** esophageal cancer, national health insurance research database, socioeconomic status

## Abstract

Esophageal cancer is the sixth leading cause of cancer mortality. More than 90% of patients with esophageal cancer in Taiwan have squamous cell carcinoma. Survival of such patients is related to socioeconomic status (SES). We studied the association between SES (individual and neighborhood) and the survival of working-age patients with esophageal cancer in Taiwan. A population-based study was conducted of 4097 patients diagnosed with esophageal cancer between 2002 and 2006. Each was traced for 5 years or until death. Individual SES was defined by enrollee job category. Neighborhood SES was based on household income and dichotomized into advantaged or disadvantaged. Multilevel logistic regression was used to compare the survival rates by SES group after adjustment for possible confounding and risk factors. Hospital and neighborhood SES were used as random effects in multilevel logistic regression. In patients younger than 65 years, 5-year overall survival rates were worst for those with low individual SES living in disadvantaged neighborhoods. After adjustment for patient characteristics, esophageal cancer patients with high individual SES had a 39% lower risk of mortality than those with low individual SES (odds ratio 0.61, 95% confidence interval 0.48–0.77). Patients living in disadvantaged areas with high individual SES were more likely to receive surgery than those with low SES (odds ratio 1.45, 95% confidence interval 1.11–1.89). Esophageal cancer patients with low individual SES have the worst 5-year survival, even with a universal healthcare system. Public health, education, and social welfare programs should address the inequality of esophageal cancer survival.

## Abstract 2

Supplemental Digital Content is available in the text

## 1 Introduction

Esophageal cancer is a leading cause of cancer death with poor 5-year survival. Worldwide, the 482,300 new cases of esophageal cancer in 2008 accounted for 4.8% of cancer cases. Esophageal cancer-related deaths in 2008 were 406,800 worldwide, accounting for 5.37% of cancer deaths. Compared with male patients in more developed areas, the mortality risk for male patients in less developed areas is about 2-fold higher. Although esophageal cancer is 3 to 4 times more common among males than females, similar inequality exists between developed and less developed areas.^[1]^ In Taiwan, esophageal cancer ranks as the ninth highest cause of cancer-related deaths, with a mortality rate of 4.7 per 100,000.^[2]^ The 2 major histological types of esophageal cancer are squamous cell carcinoma (SCC) and adenocarcinoma. SCC is clearly linked to low socioeconomic status (SES).^[3]^ Esophageal cancer incidence and survival are related to many risk factors, both individual and public. Individual factors include age, sex, alcohol intake, smoking, genetic predisposition, individual SES,^[4]^ and curative treatment. Adenocarcinoma is related to gastroesophageal reflux disease, obesity, genetic predisposition, and Barrett esophagus. More than 90% of patients with esophageal cancer in east Asian countries have SCC, a type that also predominates in Taiwan.^[5]^

In a nationwide study in Denmark, Baastrup et al^[6]^ reported that those with lower individual SES as measured by education, income, and affiliation with the work market tend to have lower relative survival, although the confidence interval (CI) overlapped between groups. Leigh et al^[7]^ reported that patients who live in more deprived area who underwent esophagectomy due to cancer had a 1.37-fold hazard ratio (HR) of postoperative death within 30 days. In older patients with locoregional esophageal cancer, racial differences in treatment and outcomes lead to poorer rates of survival.^[8]^ Ljung et al reported sociodemographic and geographical factors in esophageal cancer mortality in a nationwide study in Sweden. There is a clear inverse relationship between educational attainment and risk of esophageal cancer. Patients who live in highly densely populated areas have an increased risk of esophageal cancer.^[9]^

The combined effect of individual and neighborhood SES on esophageal cancer survival is still not clear, especially in patients of working age. We designed a population-based study, using data from the Taiwan National Health system, to analyze the combined effect of neighborhood and individual SES in esophageal cancer survival in patients of working age.

## 2 Methods

### 2.1 Ethics statement

This study was approved by the Institutional Review Board of Buddhist Dalin Tzu Chi General Hospital, Taiwan. Review Board requirement for written informed consent was waived because all data were de-identified before analysis.

### 2.2 Database

The data for this study were collected from Taiwan's National Health Insurance Research Database for the years 2002 to 2006. This dataset is organized and managed by Taiwan's National Health Research Institutes, but collected by Taiwan's National Health Insurance Program, which has been in place in Taiwan since 1995.^[10]^ The program covers approximately 99% of the residents of Taiwan and has contracts with 97% of the medical providers. Taiwan's Bureau of National Health Insurance randomly reviews the charts of 1 per 100 ambulatory and 1 per 20 inpatient claims, and interviews patients to verify the accuracy of diagnosis.

In Taiwan, the age of esophageal cancer is younger than in Western countries. According to the Taiwan Cancer Registry reports released by the Taiwan Health Promotion Administration, Ministry of Health and Welfare, 65% of esophageal cancers were diagnosed in patients younger than 65 years,^[11]^ much younger than the US reported mean age at diagnosis of 67 years.^[3]^ According to the Labor Standards Act of Taiwan, amended 2011, the maximum age of retirement is 65 years.^[12]^

Our study cohort consisted of incidental esophageal cancer patients (International Classification of Diseases, Ninth Revision, Clinical Modification [ICD-9-CM] code 150) in Taiwan who underwent treatment in a hospital for their disease at any time between 2002 and 2006. The treatment included surgical treatment and chemoradiation.

### 2.3 Measurement

Five-year survival was the key dependent variable of interest. Cause-specific survival was not used because it was not possible to determine the specific cause of death from the registry data used. Roohan et al^[13]^ have shown, in a study adapting a clinical morbidity index for use with ICD-9-CM administrative databases, that survival models for all-cause mortality and cancer-specific mortality do not differ significantly.

The key independent variables of the current study were the interaction effects of individual SES and neighborhood SES on survival. Survival of each esophageal cancer patient was determined by linking their 2002 to 2006 mortality data with claims data for the first curative treatment for the 5 years before death. With these data, we could calculate survival rates. Patient characteristics included age, sex, geographic location, treatment modality, comorbidities, and monthly income. For surgical patients, we also recorded receipt of neoadjuvant chemoradiation and adjuvant chemoradiation. Presence of comorbidities was based on the modified Charlson Comorbidity Index Score (CCIS), a widely accepted measure for risk adjustment in administrative claims datasets.^[14]^

### 2.4 Individual-level measures

This study used enrollee category (EC), which defines workplace, as a proxy for individual SES, an important prognostic factor for cancer.^[15,16]^ Patients were classified into 1 of 3 groups: (1) high SES, defined as civil servants, full-time, or regularly paid personnel with a government affiliation, or employees of privately owned institutions; (2) moderate SES, defined as self-employed individuals, other employees, and members of the farmers’ or fishermen's association; and (3) low SES, defined as veterans, those in jobless families, and substitute service draftees.

### 2.5 Neighborhood-level SES

Neighborhood SES is a contextual factor based on neighborhood household income averages and percentages reported in Taiwan's 2001 Census. In that census, neighborhood household income was measured by township using per capita income based on 2001 tax statistics released by Taiwan's Ministry of Finance.^[17]^ We categorized neighborhoods into advantaged or disadvantaged was based on the median values: advantaged neighborhoods had higher-than-median neighborhood household incomes and disadvantaged neighborhoods had lower-than-median household incomes.^[18]^

### 2.6 Other variables

We used population density, percentage of residents with college level or higher education, percentage of residents aged >65 years, percentage of residents who were agriculture workers, and the number of physicians per 100,000 residents to categorize urbanization level of residences into 1 of 7 levels.^[19]^ Urban areas were categorized as level 1, suburban areas were subcategorized into levels 2 and 3, and rural areas subcategorized into levels 4 to 7.

Hospitals were categorized by accreditation level (medical center, regional hospital, or district hospital). The geographic regions were recorded as northern, central, southern, and eastern Taiwan.

### 2.7 Statistical analysis

All statistical operations were performed using SPSS (version 15, SPSS Inc., Chicago, IL). Pearson chi-square test was used for categorical variables (sex, level of urbanization, geographic region of residence, CCIS category, treatment modality, and tumor extent) and hospital characteristics (teaching level, ownership, and caseload). Continuous variables were analyzed by using 1-way analysis of variance (ANOVA).

The cumulative 5-year survival rates were constructed for each cohort and compared using the log-rank test. The multilevel logistic regression method was used to counter the potential clustering effect of hospital and neighborhood SES. The outcomes of different SES categories were compared after adjusting for patients’ characteristics (age, sex, CCIS, urbanization, area of residence), treatment modality (radiotherapy, chemotherapy, chemoradiotherapy), and hospital characteristics (regional, district, medical center). Low individual SES and disadvantaged neighborhood were used as the reference groups. A 2-sided *P* value (*P* < 0.05) was considered significant.

## 3 Results

### 3.1 Demographic data and clinical characteristics

A total of 4097 esophageal cancer patients younger than 65 years, who received either surgery (with or without adjuvant therapy) or nonsurgical treatment, were included in this study (Table 1). Esophageal cancer patients with low individual SES were less likely than their high individual SES counterparts to receive surgical intervention, and more likely to undergo treatment in regional and district hospitals (as opposed to medical centers).

**Table 1:**
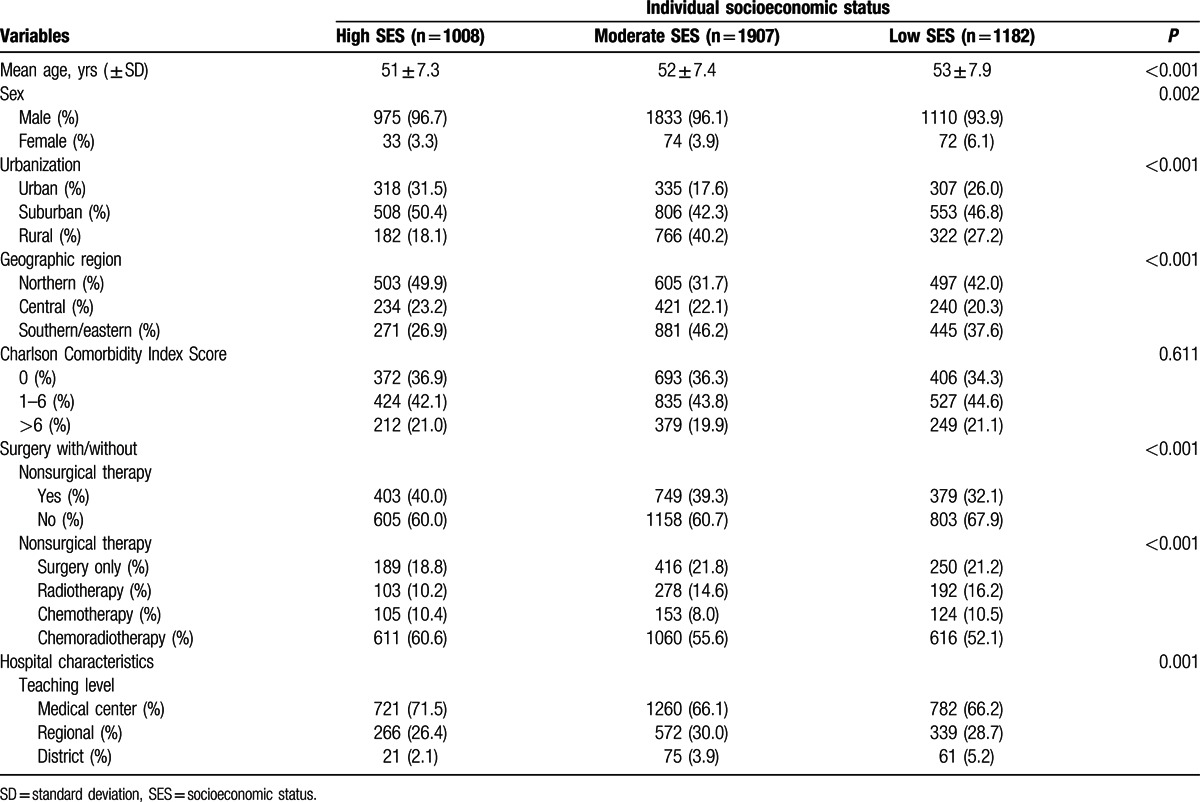
Baseline characteristics of esophageal cancer patients aged <65 years in Taiwan (2002–2006) who received treatment (n = 4097).

### 3.2 Univariate survival analysis

**Figure 1:**
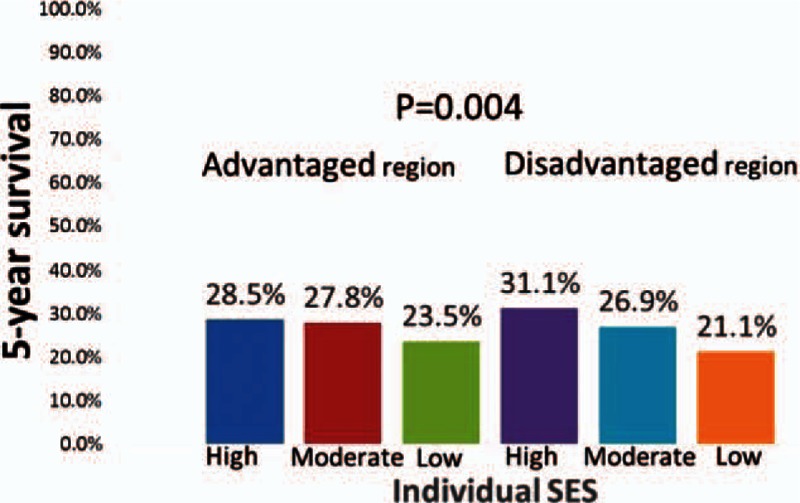
Five-year survival of esophageal cancer patients with different individual SES and neighborhood in working-age patients (n = 4097). SES = socioeconomic status.

As shown in Table 2 and Fig. 1, among the esophageal cancer patients younger than 65 years residing in disadvantaged neighborhoods, those categorized as having low SES had a significantly worse survival rate than those with high SES (*P* = 0.004). The number of patients who survived and died in each group was listed in Supplement 1.

**Table 2:**
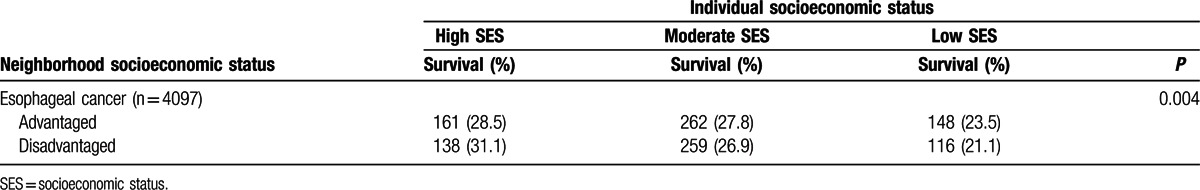
Combined effect of individual SES and neighborhood SES on 5-year overall survival rates of working-age (<65 years) esophageal cancer patients in Taiwan (n = 4097).

### 3.3 Multilevel logistic regression

The result of our univariate survival analysis indicated an interaction effect between patient age and survival rates by SES. After multilevel analysis with either hospital or neighborhood as a random effect (adjusting for age at diagnosis, sex, CCIS, urbanization, geographic region, nonsurgical therapy, and hospital characteristics), patients with low individual SES had the poorest survival. In Table 3, we listed 2 models of multilevel analysis. In the first model, the hospital was used as a random intercept, to control for the variation in medical resources, capabilities, policies, and physicians. Patients with moderate individual SES had a 29% risk reduction compared with patients with low individual SES (odds ratio [OR] 0.71, 95% CI 0.57–0.87). The patients with high individual SES had 39% risk reduction (OR 0.61, 95% CI 0.48–0.77) compared with those with low individual SES in disadvantaged neighborhoods (Table 3). There was no significant difference between patients who lived in advantaged and disadvantaged neighborhoods.

**Table 3:**
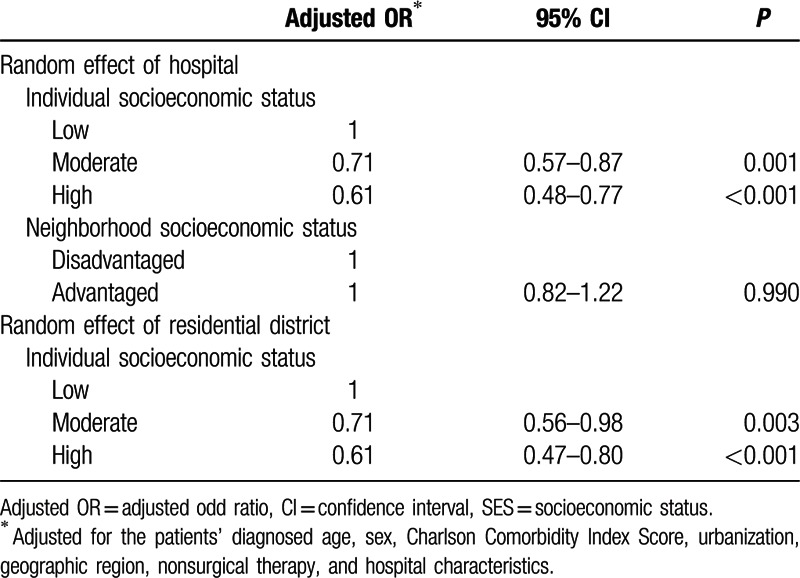
Adjusted odds ratios of individual SES and neighborhood SES for mortality by multilevel logistic regression.

In the second model, neighborhood was used as a random intercept. The patients with moderate individual SES had a 29% risk reduction compared with patients with low individual SES (OR 0.71, 95% CI 0.56–0.98). Patients with high individual SES had a 39% risk reduction (OR 0.61, 95% CI 0.47–0.80) compared with those with low individual SES (Table 3). For patients living in disadvantaged neighborhoods, those with higher SES tended to receive surgery, compared with those with low SES (OR 1.45, 95% CI 1.11–1.89) (Table 4).

**Table 4:**
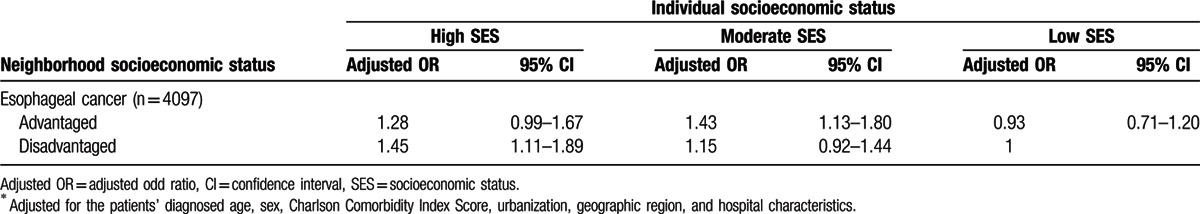
Odd ratios of individual SES for surgical treatment in advantaged and disadvantaged neighborhoods of working age (<65 years) esophageal cancer patients in Taiwan (n = 4097)^∗^.

## 4 Discussion

In Taiwan, 65% of esophageal cancer patients are under 65 years old and thus are part of the labor force. This population-based study under a system of universal health insurance included 4097 patients of working age. When we studied neighborhood and individual SES, we found that patients with low individual SES had the worst outcome, regardless of where they lived. Patients with high individual SES tended to receive surgical treatment.^[20]^

Both individual SES and neighborhood SES have been separately linked to the incidence of esophageal cancer.^[4,21]^ The prognostic factors for longer survival in esophageal cancer include localized stage, family history,^[22]^ gene expression,^[23]^ sex, age, curative treatment, higher neighborhood SES, high individual SES, treatment in university hospital, and treatment by a high-volume surgeon.^[24]^

In the literature, SES is typically presented at either the individual level or the neighborhood level. Neighborhood socioeconomic context may affect health outcomes, after adjustment for individual SES. Higher individual SES patients with higher income may receive organized and opportunistic screening for upper gastric cancer faster than lower-income groups,^[25]^ which may lead to earlier detection of cancer. Deprived neighborhood may indicate less availability of medical resources, a more polluted environment, less social support, and poorer attitudes toward health.^[26]^ Patients living in disadvantaged SES neighborhoods tend to have higher levels of social isolation, depression, and occasional stress than those who live in advantaged neighborhoods.^[27]^ In the Netherlands, neighborhood SES was found to be related to treatment choice for esophageal cancer.^[28]^ The 3-month survival rate from esophageal cancer surgery is better for patients treated by a high-volume surgeon.^[24,29]^ In population health terms, living in a disadvantaged SES neighborhood may indicate inequalities of medical resources, such as fewer hospitals and surgeons, both reported to decrease disease treatment outcomes.^[30,31]^ In patients receiving palliative treatment, high SES was associated with lower mortality, but no effect was found for patients receiving curative treatment.^[28]^ Early diagnosis and multimodality treatment of cancer improved survival for all groups, but overall mortality differed between advantaged and disadvantaged neighborhoods.^[32]^ In our study, the patients with high and moderate individual SES had lower risk for mortality than those with low individual SES.

Such observations indicate that, even in populations with universal health insurance, esophageal cancer patients with low individual SES had the worst survival rates of any cohort. Esophageal cancer patients need early detection and multimodal treatment to improve their outcomes, especially curative treatment.^[5]^ Patients from higher SES groups communicate more effectively with medical professionals to obtain health care.^[33]^ van Vliet et al^[34]^ and Bus et al^[28]^ reported that patients with high SES tend to receive curative treatment. In our study also, patients with high individual SES tended to receive surgical treatment.

This study has several limitations. One limitation is that the diagnosis of esophageal cancer, and also any other comorbidity in this study, was garnered from the ICD-9-CM codes recorded in National Health Insurance claims. Whereas this method of detection is not ideal, the National Health Insurance Bureau in Taiwan does randomly review charts and interview patients to spot-verify the accuracy of diagnosis. Another limitation was our lack of access to detailed information from the insurance claims database in terms of esophageal cancer stage, pattern of relapse, personal education, and other risk factors, such as tobacco use and dietary habits. The other important limitation is that different SES groups may differ in cancer staging and this difference may influence surgical treatment. Another factor that may influence surgery is treatment choice. However, given the robustness of the evidence and statistical analysis in this study, these limitations are unlikely to compromise our results.

Working-age patients with low individual SES have poorer outcomes than similarly aged patients with high individual SES. Patients with low individual SES need improved early detection and disease resection, greater access to medical resources, and also more health education to improve the overall outcomes for esophageal cancer. Institution of social welfare programs and national health insurance broke the financial barriers preventing poorer patients from receiving medical care, but a health gap associated with the poverty gap remains.

## Supplementary Material

Supplemental Digital Content
