# Supplementary material for: The effect of individual and neighborhood socioeconomic status on esophageal cancer survival in working-age patients in Taiwan
Source: Medicine (Baltimore). 2016 Jul 8;95(27):e4140. doi: 10.1097/MD.0000000000004140 (PMC5058858; doi:10.1097/MD.0000000000004140)
Supplement: Supplemental Digital Content [file medi-95-e4140-s001.docx]

| **Supplement 1.**  Combined effect of individual SES and neighborhood SES on 5-year overall survival of working age (<65 years) esophageal cancer patients in Taiwan (n=4,097) | | | | |
| --- | --- | --- | --- | --- |
| Neighborhood  socioeconomic status | Individual socioeconomic status | | | |
|  | High SES | Moderate SES | Low SES |  |
|  | *Survival/Total* | *Survival/Total* | *Survival/Total* |  |
| Esophageal cancer (n=4,097) |  | | |  |
| Advantaged | 161/565 | 262/943 | 148/631 |  |
| Disadvantaged | 138/444 | 259/964 | 116/550 |  |
| Abbreviations: SES, socioeconomic status. | | | | |
